# Rheumatoid arthritis reprograms circadian output pathways

**DOI:** 10.1186/s13075-019-1825-y

**Published:** 2019-02-06

**Authors:** Toryn M. Poolman, Julie Gibbs, Amy L. Walker, Suzanna Dickson, Laura Farrell, James Hensman, Alexandra C. Kendall, Robert Maidstone, Stacey Warwood, Andrew Loudon, Magnus Rattray, Ian N. Bruce, Anna Nicolaou, David W. Ray

**Affiliations:** 1 Division of Digestion, Endocrinology and Metabolism, The University of Manchester, Manchester, M13 9PT UK; 2 Laboratory for Lipidomics and Lipid Biology, Faculty of Biology, Medicine and Health, The University of Manchester, Manchester Academic Health Sciences Centre, Manchester, M13 9PT UK; 3 Specialist Medicine, Central Manchester Foundation Trust, Manchester, M13 9PL UK; 4 PROWLER.io, Cambridge, CB2 1LA UK; 5 Biological Mass Spectrometry Core Research Facility, Faculty of Biology, Medicine and Health, The University of Manchester, Manchester, M13 9PT UK; 6 Arthritis Research UK Centre for Epidemiology, Centre for Musculoskeletal Research, Faculty of Biology, Medicine and Health, The University of Manchester, Manchester, UK; 7 NIHR Manchester Musculoskeletal Biomedical Research Unit, Central Manchester University Hospitals NHS Foundation Trust, Manchester Academic Health Science Centre, Manchester, UK; 8 NIHR Oxford Biomedical Research Centre, John Radcliffe Hospital, Oxford, UK and Oxford Centre for Diabetes, Endocrinology and Metabolism, University of Oxford, Oxford, OX37LE UK

**Keywords:** Circadian, Arthritis, Ceramide, Immune cell, Rheumatoid arthritis

## Abstract

**Objective:**

We applied systems biology approaches to investigate circadian rhythmicity in rheumatoid arthritis (RA).

**Methods:**

We recruited adults (age 16–80 years old) with a clinical diagnosis of RA (active disease [DAS28 > 3.2]). Sleep profiles were determined before inpatient measurements of saliva, serum, and peripheral blood mononuclear leukocytes (PBML). Transcriptome and proteome analyses were carried out by RNA-SEQ and LC-MS/MS. Serum samples were analysed by targeted lipidomics, along with serum from mouse collagen induced-arthritis (CIA). Bioinformatic analysis identified RA-specific gene networks and rhythmic processes differing between healthy and RA.

**Results:**

RA caused greater time-of-day variation in PBML gene expression, and ex vivo stimulation identified a time-of-day-specific RA transcriptome. We found increased phospho-STAT3 in RA patients, and some targets, including phospho-ATF2, acquired time-of-day variation in RA. Serum ceramides also gained circadian rhythmicity in RA, which was also seen in mouse experimental arthritis, resulting from gain in circadian rhythmicity of hepatic ceramide synthases.

**Conclusion:**

RA drives a gain in circadian rhythmicity, both in immune cells, and systemically. The coupling of distant timing information to ceramide synthesis and joint inflammation points to a systemic re-wiring of the circadian repertoire. Circadian reprogramming in response to chronic inflammation has implications for inflammatory co-morbidities and time-of-day therapeutics.

**Electronic supplementary material:**

The online version of this article (10.1186/s13075-019-1825-y) contains supplementary material, which is available to authorized users.

## Introduction

Rheumatoid arthritis (RA) is a chronic inflammatory disease affecting approximately 1% of adults [1]. RA is associated with significant comorbidities most notably premature cardiovascular disease and insulin resistance [2, 3] and is classically rhythmic, with hallmark symptoms such as pain and stiffness typically being worse in the early morning. This has been attributed, at least in part, to underlying circadian patterns in cytokine production, including IL-6 and IL-1β [4, 5].

All human physiology is circadian; the external environment is sampled by light input through the retina, and neural transmission to the central clock in the suprachiasmatic nucleus (SCN) [6]. The core cellular clock comprises a transcription-translation feedback loop in which heterodimers of BMAL1 and CLOCK drive expression of PER, and CRY proteins in one negative feedback arm, and the two REV-ERB paralogues in the other feedback arm [7]. The period of the clock is affected by post-translational modifications of the feedback proteins, notably by phosphorylation [8].

Perturbation of the circadian clock has a significant detrimental effect on immune response, whether by genetic targeting of core clock components, or imposition of phase shifts in light-dark [9]. Indeed, shift work has been associated with risk of RA in women [10]. Although numerous lines of evidence support a role for the immune system in affecting circadian clock function [11], most observations have been made either in healthy animals or in response to acute inflammatory challenges, and there is a notable lack of studies in human subjects with prevalent, chronic inflammatory disease, such as RA.

Here, we use a systems biology approach to analyse circadian patterns of gene regulation, and protein phosphorylation in circulating immune cells from patients with rheumatoid arthritis. We selected these cells as they are accessible for serial sampling and include some of the effector cell types responsible for rheumatoid arthritis disease expression. Surprisingly, we found greater time-of-day variation in gene expression profile in RA patients than controls, a gain of rhythmic function, driven by changes in the phosphoproteome. Such an increase was also seen in serum lipid changes, particularly of the ceramide class; a gain in circadian rhythmicity was also seen in mouse experimental arthritis, driven by the acquisition of circadian rhythmicity of the ceramide synthases in the liver. Therefore, chronic joint inflammation serves as a circadian organiser, differentially coupling lipid-metabolic pathways to the core clock.

## Materials and methods

### Study design

We recruited adults (age 16–80 years old) with a clinical diagnosis of RA and age- and gender-matched controls from The Kellgren Centre for Rheumatology, Manchester University NHS Foundation Trust. All patients were seropositive for rheumatoid factor (RF) and/or anti-citrillunated peptide antibodies (ACPA), fulfilled 2010 ACR/EULAR Criteria for RA [12] and had active disease with a DAS28 > 3.2. Controls who did not suffer from any chronic inflammatory disease were recruited. Exclusion criteria in both groups were as follows: regular or recent (1 month) medication affecting sleep, glucocorticoid use, sleep disorder, BMI > 30, shift working, and recent (< 2 weeks) trans-time zone travel. The medication for each patient is shown in Additional file 1: Supplemental file S1. Two patients were on no disease modifying or biologic therapy at the time of study. One was a recently diagnosed early RA, and the other had stopped methotrexate and hydroxychloroquine and was being worked up to start a new therapy. See Additional file 2: Supplementary methods file for power calculations.

Subjects recorded a sleep diary and wore an actigraph, (PAL Technologies Ltd., UK) [13] for 1 week at home, before being admitted to the NIHR/Wellcome Trust Manchester Clinical Research Facility for a 24-h stay. Subjects were not subjected to circadian conditions, but rather were in a room with access to natural light, and no television from midnight until 7 am. Prescribed inactivity was between 10 pm and 7 am. Meals were provided as per standard MCRF protocol, at 7 pm, 8 am, and 1 pm. All subjects had free access to water. The study protocol was approved by local ethics committee (IRAS 102779, REC ref. 12/NW/0815).

### Statistical analysis

Univariate analysis was carried out using a normal data general linear model, repeated measures ANOVA or a Wilcoxon rank sum test. Normality was determined using a Q-Qplot (using qqnorm) where appropriate. Perseus version 1.6 was used to determine mean lipid levels (with a permutation-based false discovery rate). Rhythmic lipids were determined using a Gaussian process regression with a periodic kernel [14], implemented in python (using Enthought solutions). The data are normalised to have zero mean and unit standard deviation per participant and per lipid. Then, the hierarchical statistical model (based on [15]) accounts for structure in the data in the following way. The concentration of each lipid is modelled using a hidden Gaussian process, which is common to all participants in the study. This Gaussian process is given a rhythmic structure using the covariance function described by [14]. Each participant’s lipid level is modelled as a further deviation from this Gaussian process by another Gaussian process, which is lipid- and participant-specific, using a standard Matern 3/2 covariance function. Further variation is modelled as Gaussian noise. A small number of parameters controlling the relative variance of the Gaussian processes in the hierarchy are estimated by maximum likelihood.

The data likelihood ratios were generated for healthy and RA patients; 5% level (chi-squared distribution) was considered significant. Missing values (due to one broken vial) were imputed with the MICE package in R [16]. There were missing values for healthy subject 8 (due to inadequate morning blood collection) and saliva from RA subject 3. RA10 left the study early and was replaced by RA11. Gene ontology analysis was carried out using PANTHER v11 [17] .

### RNAseq

RNA integrity was determined using a Tape Station (Thermo); no loss of RNA integrity was observed during storage. Four samples were run per lane on a HiSeq (Illumina). Sequencing data were processed with Bowtie-TopHat (with Hg38). Differential expression was determined using EdgeR/LIMMA in the R environment. EdgeR implements generalised linear models. Using this approach, we were able to perform within- and between-group differences with a strict control over the error rate.

### Proteomics and phosphoproteomics

See Additional file 2: Supplementary methods file.

### Mediator lipidomics

Mediator lipidomics was performed using targeted quantitative LC-MS/MS assays [18–20]. See Additional file 2: Supplementary methods file.

### CIA mouse model

All animal procedures were performed in accordance with the United Kingdom Animals (Scientific Procedure) Act 1986. Animals were housed in 12:12 lighting conditions (LD; 12-h light to 12-h dark) with ad libitum access to normal rodent chow. All times are Zeitgeber (ZT), where ZT0 is lights on.

Male DBA/1 mice (Envigo) age 8–12 weeks were immunised with 1 mg/ml bovine type II collagen (MD Bioscience, Zurich, Switzerland) emulsified with an equal volume of complete Freund’s adjuvant (CFA; MD). The emulsion (50 μL) was injected intradermally, under anaesthesia in two sites just above the base of the tail. As a control, animals were injected with an emulsion of saline and CFA in equal volumes. Twenty-one days later, a booster (200 μL 1 mg/ml collagen in saline) was administered intraperitoneally to CIA animals. Immunisation and boost were routinely administered at zeitgeber time (ZT) 6. Disease incidence and severity and hind paw thickness were monitored from 18 days post immunisation (DPI). Disease severity was scored in each limb on a 4-point scale: (1) one inflamed digit, (2) two or more inflamed digits, (3) swelling of the foot pad and minor ankylosis and (4) severe swelling of the foot pad and joint and severe ankylosis, and the sum was calculated. RNA was extracted using phenol-chloroform (limbs) or a SV RNA extraction kit (liver) (Promega). First strand synthesis was carried out using a GoScript cDNA conversion kit; qPCR was carried out using GoTaq qPCR master mix (see Additional file 2: Supplemental methods for primer sequences).

## Results

### Assessment of parameters of diurnal activity in RA patients

**Fig. 1:**
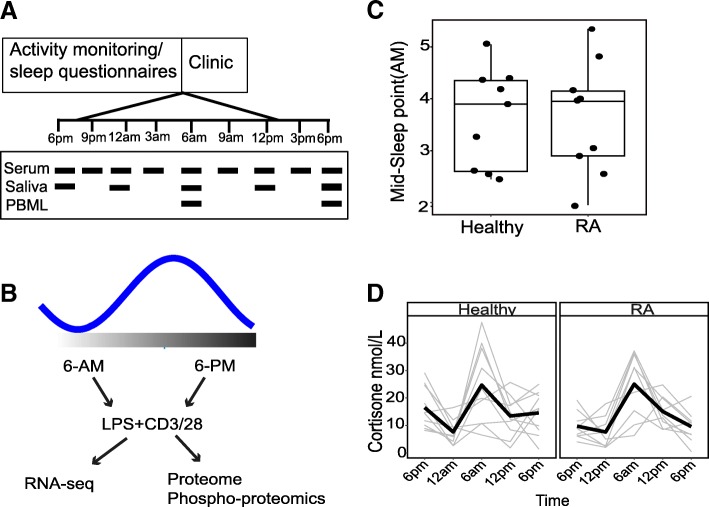
**a** Study design. Ten healthy and 10 RA patients were monitored for 1 week with an activity device, before a 24-h period in a clinical research facility. **b** PBMLs were isolated at 6 am and 6 pm; cells were stimulated with LPS/anti-CD3/28 mix for 2 h. **c** Mid sleep points were calculated for each subject. **d** Salivary cortisone concentration in healthy (*n* = 10) subjects and RA patients (*n* = 9). Graphs show the change in concentration over time. Individuals are tracked through time, and the group mean is plotted as a thick black line

We recruited patients with seropositive RA and age-/gender-matched healthy controls and examined their sleep profile for 1 week, before inpatient measurements of saliva, serum, and peripheral blood mononuclear leukocytes (PBML) (Fig. 1a, b). Our RA (*n* = 10) cohort was selected on the basis of active disease, DAS28 score > 3.2, but without recent steroid use (Additional file 1: Supplemental file S1 and Additional file 2: Supplementary methods for power analysis and study design). We did not detect any group differences in sleep metrics, or hypothalamic-pituitary-adrenal axis function or rhythm (Fig. 1c, d, Additional file 3: Figure S1A-E).

### Identification of diurnal gene expression changes specific to RA patients

To obtain sufficient cells for analysis, as we were limited in sampling frequency, we selected dawn and dusk (06:00 and 18:00); these time points had previously been found to show the greatest difference in multiple animal models of circadian control of inflammation [21]. Additionally, the peak of clinical RA disease activity is at 06:00 [22] at the end of the rest phase.

**Fig. 2:**
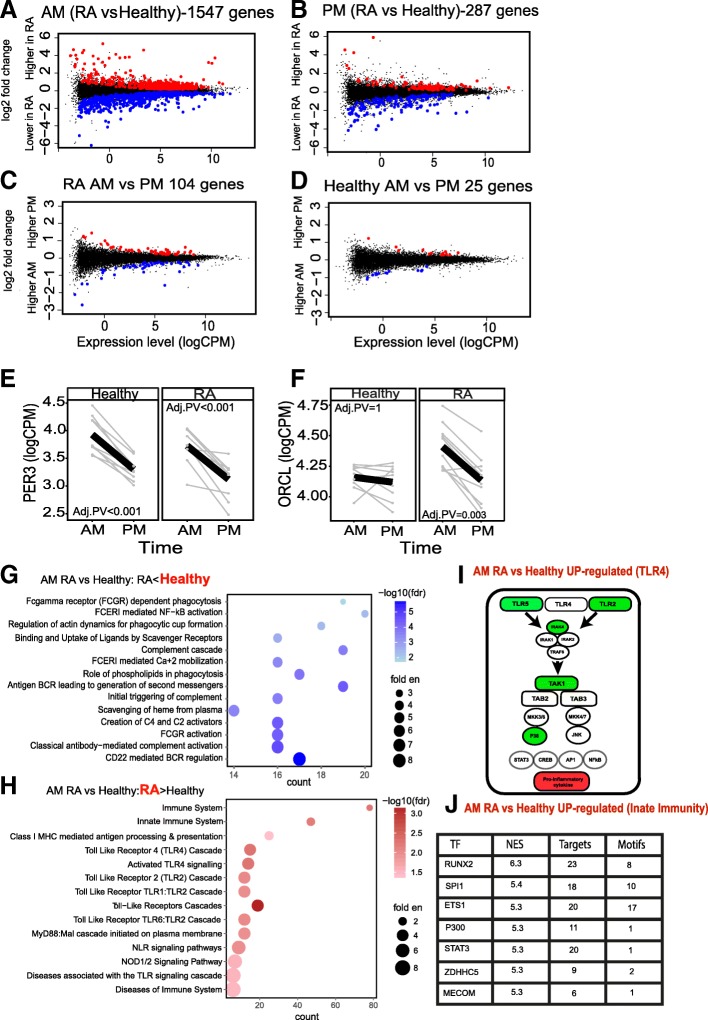
RNA-SEQ analysis of PBMLs isolated from RA patients and healthy subjects at 6 am and 6 pm. **a**, **b** Mean-difference (MD) plots for gene expression differences between RA (*n* = 10) and healthy (*n* = 9) subjects at **a** 6 am and **b** 6 pm. **c**, **d** MD plots showing differences by time of day within each group, **c** RA and **d** healthy subjects (using the model ~time + subject). Logarithm of counts per million reads (LogCPM) was calculated for each subject, and the mean line is shown in black. **e** Expression of the circadian clock gene *PER3* was determined from RNA-SEQ data. Some genes only changed by time of day in RA, **f** ORCL. Comparisons were made using Edge R glmLRT, and exact *p* values are shown (FDR corrected). Differentially expressed genes between healthy and RA at 6 am were tested for enrichment using the PANTHER/Reactome database (a selection is shown), genes that were lower in RA at 6 am (**g**) or higher in RA at 6 am (**h**)**. i** Reactome pathway analysis of the toll-like receptor 4 (TLR4) cascade network discovered in **h** is represented. Green showing 6 am upregulated genes, specifically of the TLR signalling pathway (**j**) The innate immune system genes were analysed by iRegulon to identify enriched transcription factor binding, based on the TRANSFAC database and ENCODE (using iRegulon). Normalised enrichment scores (NES) indicate a motif that covers a large proportion of the input genes (> 3, which corresponds to an FDR between 3 and 9%)

Comparing gene expression between RA patients and healthy controls, we found 1547 genes (Fig. 2a) to be differentially expressed at 06:00, but in marked contrast, only 287 genes (Fig. 2b) at 18:00, emphasising the magnitude of the circadian effect. The overlap between these two datasets is shown in Additional file 4: Figure S2E. There was no effect of age, BMI or the salivary concentration of cortisone on differential gene expression at these time points (using linear regression of differentially expressed genes against age, BMI or 06:00/18:00 salivary cortisone concentrations). Genes identified as significant in these comparisons showed no correlation with gene expression level. Within the RA group, we identified 104 genes differing between AM and PM, but only 25 in healthy controls, suggesting a gain in rhythmic gene with disease (Fig. 2c, d), which was not due to differences in circulating immune cell repertoire (Additional file 4: Figure S2A-D).

PER3 is a robust circadian gene in humans [23], and this showed similar time-of-day changes in both RA and control groups, peaking in the morning (Fig. 2e), indicating that the immune cell core clock operation was likely unaffected by disease and that the study conditions did not significantly perturb underlying circadian rhythmicity. Some genes gained a time-of-day variation in expression with RA, such as OCRL (Fig. 2f), and also included IL6ST, SOCS3, TLR2 and HCAR3 (shown in Additional file 4: Figure S2F-I), again supporting gain of rhythmic function in disease. We also found a number of genes whose expression was determined by disease status, but not responsive to time of day, LTBR4 and TNFRSF13B (Additional file 4: Figure S2J, K).

Analysis of the differentially expressed genes at 06:00 revealed enrichment of terms implicating phagocytosis and antibody-mediated complement activation and B cell activation pathways (Fig. 2g) and immune cell activation, particularly TLR pathways and innate immune responses (Fig. 2h and example of TL4 cascade genes shown in Additional file 4: Figure S2L and M). Figure 2i shows the expanded innate immune network from Fig. 2h, which includes MAPK14 (P38a). Likely, transcription factors involved were SP1 and STAT3 (Fig. 2j). Although fewer genes were differentially expressed at 18:00, similar Reactome pathways were identified.

### Integrated analysis of the HPA axis and PBML gene expression

The connections between the core cellular circadian pacemaker, the HPA axis (CORT) and the HPA axis biomarker (*GILZ)* were investigated further using non-parametric analytical approaches (Additional file 5: Figure S5B, C). In all subjects, robust correlations between the core clock genes were seen, as a positive association between *GILZ* and salivary cortisone, as expected. The relationships between core clock gene expression were all stronger in the RA group compared to the controls (depicted by diameter of the circles in the plot), again strongly supporting the emergence of a more robust, more tightly cross-coupled circadian oscillator in the presence of chronic, active inflammation (Additional file 5: Figure S5B and C).

### Identification of diurnal proteome changes in RA

**Fig. 3:**
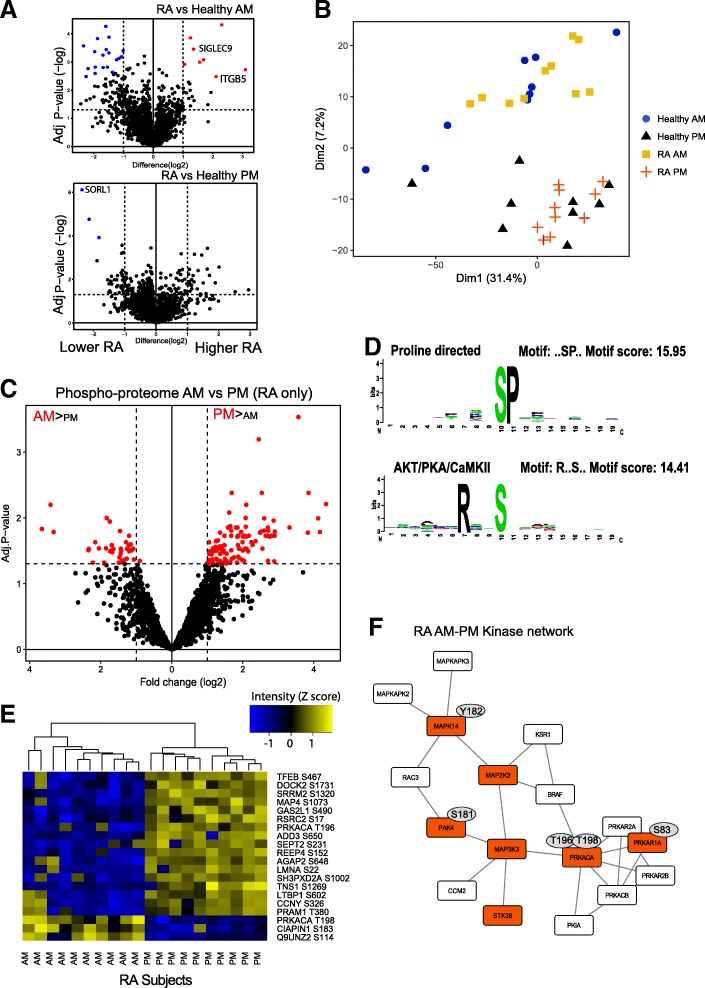
Proteome and phosphoproteome analysis in PBML. (**a**) Proteome analysis of PBMCs at 6 am and 6 pm, significant proteins were determined using a t-test with permutation FDR. **b** PCA analysis of phosphosites at 6 am and 6 pm in RA and healthy subjects. **c** Volcano plot of the significant sites differing between 6 pm and 6 am in RA; significant sites were determined using the LIMMA package. **d** Sequence logo of significant sites between 06:00 and 18:00 found in RA. **e** Heat map showing the most significant time-of-day-regulated sites in RA only (peptide intensity levels shown [*Z* score]. Samples were scaled by row; clustered by row and column). **f** Kinase network in RA. Kinases with phosphosites regulated by time of day in RA only were analysed in String (V10) and cytoscape. Red nodes indicate identification from dataset and white nodes were inferred by the String (V10) software. Known regulatory sites are shown in grey

Because we observed such striking differences in gene expression at 06:00, we measured the phosphoproteome in pursuit of upstream causative pathways. The total immune cell proteome was analysed at 06:00 and 18:00, and we found changes between healthy and RA patients with the greatest difference again being at 06:00 (Fig. 3a). Of the 2710 proteins detected, we found only 27 that were increased in RA. At 18:00, only 3 proteins differed in expression between RA and controls.

Protein determination thus identified relatively few changes dependent on disease state. In marked contrast, phosphoproteomic profiling revealed extensive differences, with the striking observation showing clear separation between dawn and dusk specific to RA patients (Fig. 3b). This was a similar gain of temporal effect as was seen with the earlier transcriptome analysis (Fig. 2c, d).

A total of 147 phosphosites varied between dawn and dusk in the RA group (Fig. 3c) from which we identified a consensus arginine and proline directed phosphoserine site as strongly enriched within the temporally controlled RA phosphoproteome (Fig. 3d). Interestingly, some sites such as phospho-T198 of cAMP-dependent protein kinase alpha (PRKACA, PKAa) were higher in the morning, while a closely related site phospho-T196 was higher in the evening. Phospho-sites that were unique to RA included HSP27-S82, MAPK14(p38)-Y182 and cAMP-dependent PKA-T198 (Fig. 3e). The time-of-day-regulated kinases formed a functional network (Fig. 3f), with phosphosites found to differ by time of day and to have known regulatory function marked on the figure, with inferred connecting nodes as white. This analysis revealed multiple members of the MAP kinase family, and PKA, as being regulated in RA cells by time of day.

### Immune cells responses to activation by time of day in RA

**Fig. 4:**
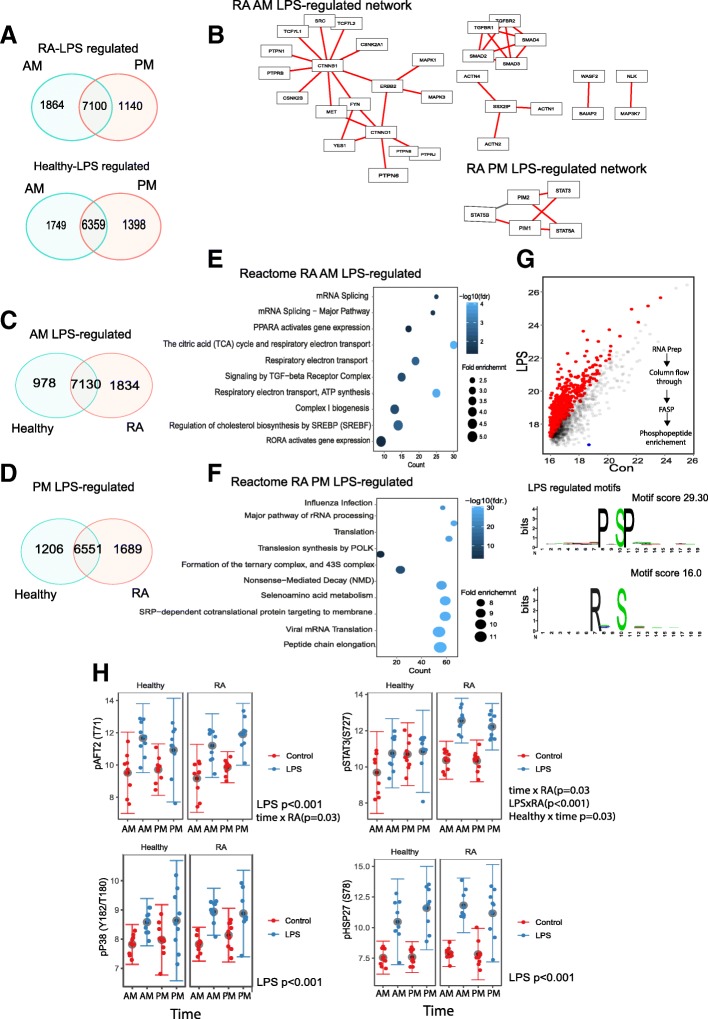
Time-of-day immune cell activation. PBMLs were isolated at 6 am or 6 pm and stimulated for 2 h with LPS/anti-CD3/28. Then mRNA was purified and subject to RNA-SEQ analysis. **a** Venn diagrams were generated using all the significantly expressed genes in each comparison. **b** Genes that were significantly regulated by LPS at either time point in RA were subjected to Edge set analysis (pathway-result network diagram shown). The Edges that contribute to the Edge set are labelled in red. **c** AM and **d** PM LPS-regulated genes were compared (healthy vs RA) (shown as Venn diagrams). Genes that were unique to RA at each time point (from **c** and **d**) were analysed using the Reactome database (AM genes (**e**) and PM genes (**f**)). **g** LPS-stimulated phospho-proteome analysis. Significant sites are shown in red (unregulated by LPS and blue (downregulated by LPS). Motif analysis of LPS-regulated sites shown below. **h** Luminex assay for phospho-p38, HSP27, STAT3, ATF2 and fluorescence values was log transformed (mean and SD shown) and analysed using a repeated measures ANOVA

As we observed major changes in basal gene expression and phospho-proteome in immune cells from RA patients at dawn, we sought responses to cell activation ex vivo. Circulating immune cells were activated (with LPS and anti-CD3/28; from here LPS denotes the mixture of stimuli). This revealed more than 8000 transcripts regulated by LPS (Fig. 4a). We further analysed differentially regulated genes using Edge set enrichment analysis (using healthy AM as the control character and genes activated in RA at both times), showing several well-characterised disease targets as being implicated in the differential pattern of gene expression (Fig. 4b). This, again, included members of the MAP kinase family (MAPK1, MAPK3, MAP3K7). In the PM samples, STAT3 activity was inferred (Fig. 4b: see Additional file 6: Figure S3A and B for full networks). Pathway analysis of genes regulated in only in RA at 06:00 and 18:00 implicated AKT/PTEN (06:00) and RNA polymerase components (18:00) (Additional file 6: Figure S3C,D).

More genes responded to activation in RA (Fig. 4c, d). Analysis of RA-specific responder genes at 06:00 (Fig. 4e) or 18:00 (Fig. 4f) highlighted metabolic pathways and translational control respectively. Analysis of likely transcriptional regulators of 06:00 and 18:00 LPS-regulated genes in RA identified a number of candidate transcription factors, including RXRa (06:00) and CREB1 (18:00) (Additional file 7: Figure S4A-C), both targets for PKA, which we find is time of day regulated in RA (Fig. 3f).

Phosphoproteomic analysis identified 1494 phosphorylated motifs in total, of which 664 were regulated by cellular activation (Fig. 4g, Additional file 8: Supplemental data file). Phosphorylation sites on HSP27 (S15, 65 and 82) and STAT3 (S727 and Y705) were highly responsive to LPS, but in these ex vivo studies, we did not detect any time-of-day difference in response. Motif analysis highlighted a role for MAPK (PxSPx) and AKT/PKA (RxxS), a similar pattern to that previously found (in Fig. 3d); however, the PKAa T198 site that we previously found to be regulated by time of day (Fig. 4g) was unresponsive to LPS.

To quantify more precisely changes in phosphorylation of these and other members of the MAP kinase-signalling network, we moved to a quantitative antibody-based bead array method (Luminex) (Fig. 4h). Phospho-STAT3(S727) was increased by LPS and was found higher in RA (*p* = 0.005). There was also a disease and time interaction (*p* = 0.03), with STAT3 phosphorylation increased at 18:00 in the healthy controls. ATF2, a transcription factor and target for ERK1/2 (and other MAPKs), was phosphorylated in response to LPS (T71) (*p* < 0.001) and showed a time-of-day increase in the RA group (disease state × time, *p* = 0.03), further adding to the previous finding that MAPK signalling is altered in the RA group by time of day.

### Serum lipidomic analysis in RA patients reveals newly rhythmic ceramide species

In additional to regulating mRNA expression, circadian rhythms also regulate the concentration of other biomolecules, including circulating serum components. As metabolite circadian rhythms can act on clock gene circuits to affect their function, and in turn clock output pathways can regulate the metabolome, we also analysed the serum metabolome in the same two groups of subjects [24–26]. In previous studies in healthy human volunteers, the major circulating metabolome components lying under circadian control were lipids, many of which have well-defined signalling functions in inflammation and immunity [27]. Therefore, we focussed our attention on serum lipids capable of transmitting signals to target cells.

**Fig. 5:**
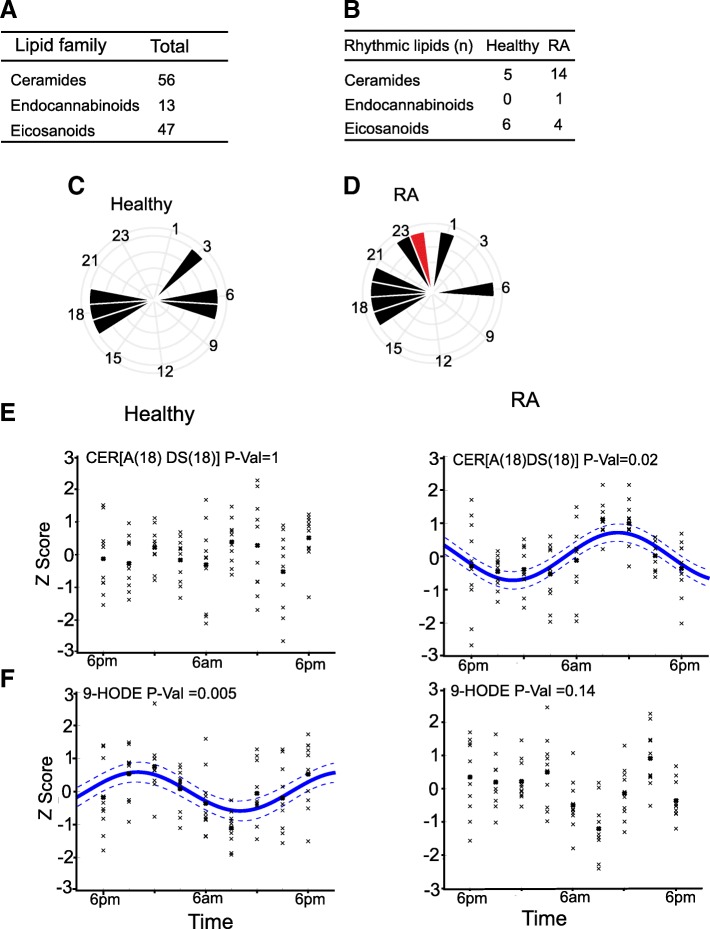
Identification of rhythmic serum lipid species in RA patients. **a** Numbers of serum lipid species detected in the three classes. **b** Number of rhythmic lipid species by class identified in healthy and RA subjects, using a Gaussian process model. **c**, **d** The estimated time of peak serum concentration of each rhythmic serum lipid species in healthy and RA. Ceramide peak (pooled CER[NDS]) shown in red. **e** Circadian profile of CER[A(18)DS(18)] and **f** 9-HODE in healthy subjects (*n* = 10) and RA patients (*n* = 10) respectively. Rhythmicity tested by Gaussian process. *P* value indicates degree of significance; trend lines included for significant oscillations only**.** The posterior standard deviation of the mean Gaussian process is shown by the dotted line

We used a targeted LC-MS/MS approach and measured 116 ceramides, eicosanoids and endocannabinoids (Fig. 5a). The ceramides were further classified according to structure, comprising either a sphingosine (S) or dihydrosphingosine (DS) sphingoid base and either a non-hydroxy (N) or alpha-hydroxy (A) fatty acid. Overall, we could not distinguish study groups by PCA analysis of serum lipids (not shown), and there was no effect of gender, age, or BMI (using regression analysis, *p* > 0.05).

A Gaussian process model was used to identify rhythmic lipids [14, 15]. We found more rhythmic lipids in the RA group, particularly ceramides (Fig. 5b). The peak times for each rhythmic species were also estimated (Fig. 5c, d), with the acrophase of the newly rhythmic ceramides occurring at 23:00. The majority of rhythmic lipids in both healthy and RA subjects peaked at 06:00 or 18:00 (Fig. 5c, d). This suggested that the newly rhythmic ceramides were products of a newly rhythmic enzymatic pathway.

A small number of serum lipids were found to be rhythmic in both RA patients and controls, including 9HOTrE, 12(13)EpOME, 13HOTrE and the ceramides CER[N(18)DS(24)], CER[N(18)DS(26)], CER[N(22)DS(18)], CER[N(24)DS(18)] and CER[N(29)S(18)]. The newly rhythmic ceramide species in RA were particularly of the CER[NDS] class (examples shown in Additional file 5: Figure S5A), with fewer rhythmic alpha-hydroxy ceramides (CER[AS(18)DS(18)] shown as an example in Fig. 5e). Some lipids showed a stronger and significant oscillation only in healthy subjects (9-HODE shown as an example in Fig. 5f), interesting as both 9- and 13-HODE species had previously been identified as rhythmic in a healthy human circadian study [27, 28], usefully providing an external quality control for our clinical protocol.

### Serum ceramides similarly acquire a new circadian rhythmicity in mouse experimental arthritis (CIA)

**Fig. 6:**
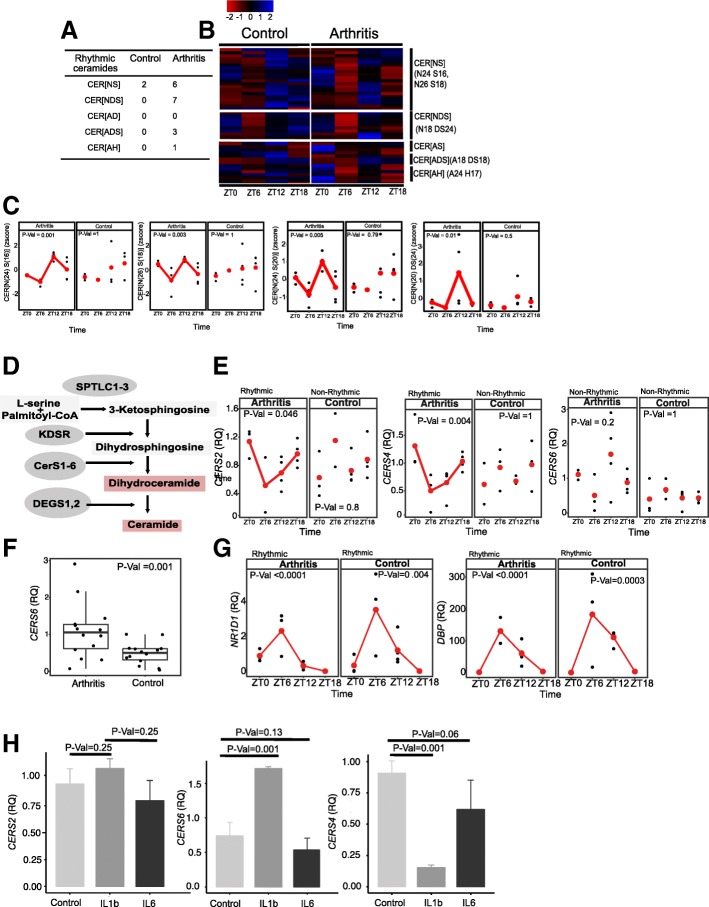
Identification of rhythmic serum ceramide species in collagen-induced arthritis (CIA) model in mouse. Serum samples were collected from CIA and control mice at the indicated times; time is Zeitgeber with ZT0 lights on and ZT12 lights off. **a** JTK cycle was used to estimate rhythmic species in control or CIA mice, and the number of ceramides in each class is recorded. **b** Heatmap of all detected serum ceramide concentrations in different classes in both CIA and control mice (mean line shown) for each group shown, examples of rhythmic species shown on the right). **c** Time course of index ceramide concentrations in CIA and control mice, CER[N(24)S(16)], CER[N(26)S(18)], CER[N(24)DS(20)] and CER[N(24)S(20)]. Adjusted *p* values are shown (*n* = 12 control and 15 arthritis). Ceramide synthase gene expression in livers from CIA and control mice. **d** Pathway map of ceramide species generation. Serine palmitoyltransferase long chain base subunit 1 (SPLTC1,2), dihydroceramide desaturase 1 (DEGS1), ketodihydrosphingosine reductase (KDSR), ceramide synthase 1–6 (CERS). **e** Livers from CIA or control mice were collected at the indicated times (ZT0–18), and RNA extracted. Ceramide synthase gene expression was determined by qPCR for CERS2, CERS4 and CERS6 at each time point. JTK cycle was used to determine rhythmicity, adjusted *p* values shown (*n* = 14 control and 14 arthritis). **f** Gene expression for CERS6 in liver from arthritis and control mice (*n* = 14). **g** NR1D1 (Rev-erbα) and DBP circadian gene expression in liver. **h** Mouse liver cells (AML12) were stimulated with IL-6 (25 ng/mL) or IL-1β (25 ng/mL) for 4 h. Ceramide synthase expression level determined for CERS2, CERS4 and CERS6 by qRT-PCR. Changes in expression were determined by a Wilcoxon rank sum test (*n* = 3)

To identify if a similar acquisition of ceramide rhythmicity was a consistent feature of chronic inflammatory arthritis, we assessed a CIA mouse model (Additional file 9: Figure S6). This model results in chronic, destructive arthritis (Additional file 9: Figure S6A,B,C), and the disease expression, measured by paw swelling or cytokine expression, shows a strong diurnal variation in severity, as in human RA [21, 29]. Mice were kept in 12:12 L:D conditions, and times are expressed at Zeitgeber time (ZT) where ZT0 is lights on or 06:00. We identified the gain of circadian oscillation in similar ceramide species in the CIA mouse serum as in RA (Fig. 6a, b). Examples of newly rhythmic ceramide species in the CIA mice are shown in Fig. 6 c.

Ceramide synthesis is complex [30] (Fig. 6d, de novo pathway shown). A number of ceramide synthase genes were found to be expressed in the inflamed limb (Additional file 10: Figure S7A-E), but while some (*CERS2*) were suppressed by inflammation, and others (*CERS4*, *5*, *6*) were stimulated, there was no variation in expression over time. Gene expression of other enzymes regulating ceramide biosynthesis did not vary by time in CIA or control mouse limbs (Additional file 10: Figure S7F).

In contrast, in the liver, we identified cycling ceramide synthase gene expression only in CIA mice. *CERS2* and *CERS4* reached their nadir at ZT6 (Fig. 6e), while *CERS6* did not oscillate but was significantly higher in the arthritic mouse liver tissue (Fig. 6f). Importantly, there was no impact of arthritis on liver core clock gene oscillation (Fig. 6g).

In order to explore mechanisms by which CIA might drive cycling hepatic ceramide synthesis, we investigated whether inflammatory mediators from the inflamed joints were acting on the liver to drive rhythmic gene expression changes over time. In the CIA model, serum IL-1β and IL-6 show circadian variation [21] and these were chosen as candidate mediators. IL-6 did not affect mouse liver cell *CERS* expression (Fig. 6h), but IL-1β potently suppressed *CERS4* and stimulated *CERS6* (Fig. 6h). Therefore, circadian production of inflammatory cytokines may convey a timing signature to hepatic ceramide synthase expression. The long-chain ceramides we see varying by time of day are principally regulated by *CERS2* and *CERS4*, both of which cycle in the liver, rather than *CERS6* which did not significantly vary across time. It is also notable that serum IL-1β peaks at ZT6 [21], the nadir of *CERS4* gene expression, and serum ceramide concentrations (Fig. 6c, e).

## Discussion

A characteristic feature of RA is the change in disease activity over the day, measured by symptoms, and circulating markers of the inflammatory process such as IL-6 [31, 32]. Indeed, there have been attempts to add circadian logic to therapy, with modified release prednisolone tablets entering the clinic [33]. Embedding circadian logic into drug development, and biomarker studies of RA, requires an understanding of the circadian contribution to disease pathogenesis and expression, as an oscillating drug target phase may not be obvious from patient-reported symptoms.

We identified preserved behavioural, endocrine and immune cell circadian rhythmicity in patients with active RA. RA patients showed far more differences in gene expression and phosphopeptide abundance by time of day than controls. This difference was essentially lost in cells cultured ex vivo, implying the presence of a strong endogenous circadian entraining factor. Such molecules have been proposed to circulate in serum, but currently their identity remains unknown [34, 35].

In the RA group, we identified major gene ontology groups involved in immune response as being regulated by time of day within the circulating immune cell compartment. Among these, there was strong enrichment for TLR4 signalling components. Analysing the genes differentially regulated at dawn between RA and control, we identified the enrichment for transcription factor binding motifs including STAT3, which is an important signalling mediator of IL-6 action. Further analysis of the immune cell phosphoproteome identified the separation of profiles by time of day only in the RA group, and among the phosphopeptides with a time-of-day signature, we inferred proline-directly serine phosphorylation, which marks MAP kinase action, and arginine-associated serine phosphorylation, which indicates PKA action. Further analysis of the phosphopeptides themselves identified changes in protein kinase phosphorylation, including multiple members of the MAP kinase family, and an interesting reciprocal phosphorylation site on protein kinase A (PRKACA T196/198). Involvement of the MAP kinases in time-of-day responses within the immune cell population may explain the enrichment of STAT3-binding sites within time-of-day-regulated genes, as MAP kinases phosphorylate STAT3. These data identify a MAP kinase-STAT3 circuit as time of day regulated in patients with active RA.

To extend our time-of-day observations, we analysed cellular responses to ex vivo activation. Here we found that the time-of-day effects were largely lost, but disease-specific differences remained. The inferred signalling networks activated included those served by multiple members of the MAP kinase family, again, and both STAT3 and STAT5. As the time-of-day differences were not seen in the ex vivo cell studies, we confined the analysis of disease-specific changes. Here, we identified a major change in the MAP kinase phosphosite on STAT3 (S727), again finding a disease-specific role for this transcription factor, a target of the IL-6-directed therapy in RA.

Ceramides play a critical role in regulating inflammation and may be produced locally within foci of inflammation, or elsewhere (e.g. the liver). In our CIA mouse model, we found similar patterns of rhythm serum ceramides as emerged in human RA. The CIA mouse model offers the advantage of no drug exposures, and controlled whole-life environmental exposures, thereby greatly increasing confidence that the ceramides result from the inflammatory process. Conservation of the newly rhythmic ceramides in RA would suggest functional importance in response to chronic inflammation. Ceramides have emerged as important drivers of insulin resistance in type II diabetes [36, 37]. Their emergence here as coupled to the circadian clock in RA suggests a possible role mediating the adverse metabolic profile associated with accelerated atherosclerosis in RA [38, 39].

We also found oscillating ceramide synthases surprisingly in the liver, and not in the joint, suggesting the liver, or other organs such as the gut [40], as the origin for the rhythmic ceramides and thereby identifying a systemic coupling of ceramide metabolism to joint inflammation. Further analysis showed that IL-1β, a rhythmically expressed cytokine in CIA [21], with peak serum concentration at ZT6, inhibits hepatic *CERS4*, resulting in a near coincident nadir in serum *CERS4* product ceramide concentration. A similar circadian organising effect of disease was reported for lung cancer, mediated by IL-6 [34], or in response to changes in the gut microbiome, mediated by polyamine metabolites [41]. Therefore, our data show that in human chronic inflammatory disease, circadian mechanisms are co-opted to establish a new homeostatic oscillatory state, but whether this is a disease-sustaining step remains to be determined.

## Conclusion

Our findings identify the emergence of enhanced circadian rhythmicity and expansion of the repertoire of rhythmic processes in the presence of chronic joint inflammation, and we demonstrate that the observations in human disease are also seen in highly controlled experimental mouse studies. The exciting discovery of distant transmission of timing information to couple ceramide synthesis to joint inflammation, through cycling inflammatory cytokine synthesis and secretion, such as IL-1β [21], points to a systemic re-wiring of the circadian repertoire in response to chronic inflammation, with implications for inflammatory co-morbidities and therapeutics.

## Additional files

Additional file 1: Supplemental File S1 RA medication history. (PDF 21 kb)

Additional file 2: Supplemental methods file. (DOCX 162 kb)

Additional file 3: **Figure S1.** Assessment of parameters of diurnal activity in RA patients. (A) Summary activity data for both healthy (*n* = 10) and RA patients (*n* = 10) was used to estimate the mid-rest phase which is plotted by clock time. (B) Salivary cortisol or cortisone levels were measured in healthy subjects (*n* = 9) and RA patients (*n* = 9) over 5 time points (from 6 pm at 6 hourly intervals). **(**D and E) Data expressed as area under the curve. (PDF 137 kb)

Additional file 4: **Figure S2.** Analysis of global gene expression in PBMLs from RA patients and healthy subjects. (A-D) Non-logged normalised CPM values were used for the analysis of PBMC cell populations by CIBERSORT. Estimated % of cell type for each subject (A) T lymphocytes, (B) NK cells, (C) Mast cells, and (D) B lymphocytes showed small differences between groups based on disease state (healthy or RA), or time of sampling (6 am or 6 pm). Significance was determined with a repeated measures ANOVA, significant *p* values for the overall effect of time (AM and PM), or state (Healthy or RA) regardless of time are shown (RA *n* = 10, healthy *n* = 9). (E) Venn diagrams were generated using all the significantly expressed genes in RA vs Healthy AM and RA vs Healthy PM. (F–K) Example genes from time-of-day RNA-SEQ analysis. Significant genes (AM RA vs Healthy) enriched in the Reactome pathway TL4 cascade are show as a table (with output from EdgeR) (L) and heatmap of expression level for each sample (M) (Expression level shown [Z score]. Samples were scaled by column; clustered by row and column.) (PDF 457 kb)

Additional file 5: **Figure S5.** (A) Healthy and (B) RA correlation analysis of circadian gene expression, and outputs of the circadian clock. The difference in gene expression (between 6 am and 6 pm) was calculated for each subject or patient, to estimate amplitude of oscillation. The difference between morning and evening salivary cortisone to determine circadian output pathway activity, and *GILZ* gene expression as a biomarker of cortisol action were also included in the correlation matrix. The circle diameter estimates strength of correlation, and the colour (red negative, and blue positive) the direction of correlation. (C) Circadian profile of CER[N(25)S(20)]in healthy and RA subjects (as identified in using a Gaussian process model). (PDF 290 kb)

Additional file 6: **Figure S3.** Pathway analysis of AM and PM LPS regulated genes. Full networks for Fig. 4b. AM-RA LPS regulated genes were used to create network graphs using Edge set enrichment analysis (ESEA)**. (**B) As (A) with genes regulated at 18:00. (C) Reactome pathways for 06:00 (LPS regulated Healthy vs RA) and (D) 18:00 (LPS regulated Healthy vs RA shown). (PDF 223 kb)

Additional file 7: **Figure S4.** Transcriptional regulators of AM and PM LPS regulated genes in RA. AM and PM LPS regulated genes were analysed using iRegulon (Cytoscape) A and B) Net enrichment scores were (NETs) were used to order the potential transcription factors. (C) Example motifs for the most significantly enriched targets identified in A and B. Normalised enrichment scores (NES) indicate a motif that covers a large proportion of the input genes (> 3, which corresponding to an FDR between 3 and 9%). (PDF 686 kb)

Additional file 8: Supplemental data file. (XLS 122 kb)

Additional file 9: **Figure S6.** Time course of arthritis induction in response to collagen immunisation in mice. The average arthritis score (A), incidence (B), and average hind paw thickness (C) were plotted against time following immunisation. Mean and standard error of the mean are shown. (PDF 183 kb)

Additional file 10: **Figure S7.** Ceramide synthesis pathway gene expression analysis. (A-E) RNA was extracted from control and arthritic mouse limb tissue at six-hourly intervals (ZT0–18). Ceramide synthase gene expression was determined by qPCR. Mean gene expression data across time is shown as a boxplot. Changes in concentration over time were analysed by a Wilcoxon rank sum test (*n* = 14 control and 15 arthritis); exact *P* values are shown. (F) Ceramide synthetic pathway gene expression in liver and limb from CIA and control mice. Tissues were harvested at six hourly intervals as described. Gene expression for serine palmitoyltransferase long chain base subunit (*SPLTC*)1 and *SPLTC2*, dihydroceramide desaturase1 (*DEGS1*). JTK cycle was used to determine rhythmicity, adjusted *P*-values shown (control *n* = 13, arthritis *n* = 15). (PDF 796 kb)

## Abbreviations

A: Alpha-hydroxy fatty acid
ACPA: Anti-citrillunated peptide antibodies
AKT: Protein kinase B
ATF2: Activating transcription factor 2
CER: Ceramide
CFA: Complete Freund’s adjuvant
CIA: Collagen-induced arthritis
CORT: Cortisol
DEGS1: Dihydroceramide desaturase1
DPI: Days post immunisation
DS: Dihydrosphingosine sphingoid base
EpOME: Epoxy-octadecenoic acid
GILZ: Glucocorticoid-induced leucine zipper
HCAR3: Hydroxycarboxylic Acid Receptor 3
HODE: Hydroxyoctadecadienoic acid
HOTrE: Hydroxy-octadecatrienoic
Hsp27: Heat shock protein 27
IL-1β: Interleukin 1 beta
IL6ST: Interleukin 6 signal transducer
LIMMA: Linear models for microarray data
LPS: Lipopolysaccharide
MAP3K7: Mitogen-activated protein kinase kinase kinase 7
MAPK1: Mitogen-activated protein kinase 1
MAPK3: Mitogen-activated protein kinase 3
N: Non-hydroxy fatty acid
NR1D1: Nuclear Receptor Subfamily 1 Group D Member 1 or Rev-Erb alpha
OCRL: Inositol polyphosphate 5-phosphatase OCRL-1
p38 or MAPK14: p38 mitogen-activated protein kinase
PBML: Peripheral blood mononuclear leukocytes
PER3: Period circadian regulator 3
PRKACA: Protein kinase alpha
RA: Rheumatoid arthritis
RF: Rheumatoid factor
S: Sphingosine
SCN: Suprachiasmatic nucleus
SOCS3: Suppressor of cytokine signalling 3
SP1: Specificity protein 1
SPLTC1: Serine palmitoyltransferase long chain base subunit 1
SPLTC2: Serine palmitoyltransferase long chain base subunit 2
STAT3: Signal transducer and activator of transcription 3
TLR2: Toll-like receptor 2
ZT: Zeitgeber time
