# Supplementary material for: Rheumatoid arthritis reprograms circadian output pathways
Source: Arthritis Res Ther. 2019 Feb 6;21:47. doi: 10.1186/s13075-019-1825-y (PMC6366099; doi:10.1186/s13075-019-1825-y)
Supplement: Supplementary file 1 — Supplemental File S1 RA medication history. (PDF 21 kb) [file 13075_2019_1825_MOESM1_ESM.pdf]

Supplementary S1

RA001	RA002*	RA003	RA004	RA005*	RA006	RA007	RA008	RA009	RA011
methotrexate	none	viscotears	methotrexate	ibuprofen	methotrexate	methotrexate	hydroxychloroquine	methotrexate	methotrexate
folic acid		tears natural	naproxen			folic acid	naproxen	rabeprazole	abatacept
tramadol		seretide spray	losartan					aspirin	sulfasalazine
paracetamol			atorvastatin					diltiazem	pregabalin
ibuprofen			gliclazide					folic acid	ibuprofen
			sulfasalazine					perindopril	calcium & vit D
			metformin					atorvastatin	folic acid
			cod liver oil						omeprazole
			ventolin						

H001	H002	H003	H004	H005	H006	H007	H008	H009	H010
Nil	Nil	Nil	Nil	Nil	Nil	Nil	Nil	Nil	Nil

Comments

RA002*New patient studied pre-treatment initiation

RA005*recently stopped MTX and hydroxychloroquine
