# Supplementary material for: Rheumatoid arthritis reprograms circadian output pathways
Source: Arthritis Res Ther. 2019 Feb 6;21:47. doi: 10.1186/s13075-019-1825-y (PMC6366099; doi:10.1186/s13075-019-1825-y)
Supplement: Supplementary file 2 — Supplemental methods file. (DOCX 162 kb) [file 13075_2019_1825_MOESM2_ESM.docx]

# Supplementary methods Sample Size and Power

A small simulation study was used to estimate the power of the study to detect circadian changes assuming a 24h sinusoidal variation in the response. The effect size is expressed as the amplitude of the circadian variation as a proportion of the within-patient variability. Plots are shown for effect sizes of 0.6, 0.8 and 1.0 for sample sizes of 6-12. 1000 experiments were simulated for each point and a standard analysis of variance with a random effect term for individual fitted and the significance (P<0.05) determined for each sample and hence the power to detect an effect.

The individual panels show sampling schemes of 2,3,4 and 6-hourly with and without a repeat of the first time point at 24h.

1. **hourly inc 24**

Effect size

0.6

0.8

1

Power

60 70 80

90

Power

60 70 80

90

Power

60 70 80

90

Power

60 70 80

90

1. **hourly inc 24**
2. **hourly inc 24**

**6-hourly inc 24**

6 7 8 9 10 11 12

40

50

40

50

40

50

40

50

n

1. **hourly exc 24**

Power

60 70 80

90

6 7 8 9 10 11 12

n

1. **hourly exc 24**

Power

60 70 80

90

6 7 8 9 10 11 12

n

1. **hourly exc 24**

Power

60 70 80

90

6 7 8 9 10 11 12

n

**6-hourly exc 24**

Power

60 70 80

90

| 6 7 8 9 10 11 | 12 | 6 | 7 8 9 10 11 | 12 | 6 | 7 8 9 10 11 | 12 | 6 | 7 8 9 10 11 | 12 |
| --- | --- | --- | --- | --- | --- | --- | --- | --- | --- | --- |
| n |  |  | n |  |  | n |  |  | n |  |

The simulation indicates that with ~8 participants sampling at 6 hourly intervals or less will give >90% power to detect an effect size equal to the natural sample variation. With 3h sampling 8 participants gives ~80% power to detect an effect size of ~2/3. More frequent sampling is beneficial as it mitigates against the risk of

40

50

40

50

40

50

40

50

missing narrow peaks. The 24h point has the benefit of giving a direct repeat but adds little power in this scenario.

# Study design

We recruited patients with seropositive RA and age/gender matched healthy controls. A sleep diary was used to measure subjective sleep, calculating the average sleep midpoint in both groups (the midpoint between sleep onset and wakening), a similar time estimated from actigraphy data (Fig 1B,S1A). RA patients had a lower step count (RA 7408.8 + 1844.9 vs 11807.3 + 3917.8; mean,SD; p<0.05).

All volunteers were admitted to our Clinical Research Facility during the afternoon and were then subject to the same schedule. They had three isocaloric meals, and were encouraged to rest in a recumbent position from 10pm till 6am. Blood was drawn from an in-dwelling venous catheter at three hourly intervals for serum analysis, and at 06:00 and 18:00 for larger volumes of blood were taken for immune cell preparation. Saliva was collected at six-hourly intervals for LC-MS/MS analysis of salivary cortisone, to measure output from the hypothalamic-pituitary-adrenal axis (Perogamvros et al., 2010). Purified immune cells were either immediately analysed, or plated and activated with LPS and anti CD3/28 (Fig 1C). No group differences in cortisone concentration or rhythm were seen in RA (Fig. 1D,S1B-E).

# Eicosanoid analysis

Serum eicosanoids were quantified by ultraperformance liquid chromatography with electrospray ionisation and tandem mass spectrometry (UPLC/ESI-MS/MS) as previously described [1]. Briefly, serum samples (approximately 1 mL volume) were measured and diluted with 700 µL ice-cold methanol and water to a final volume of

4 mL (final concentration 15 % methanol v/v). Internal standards (20 ng each of PGB_2_-*d*4, 12-HETE-*d*8, 8(9)EET-*d*11 and 8,9DHET-*d*11 (Cayman Chemical Anna Arbor, USA)) were added and incubated on ice for 15 min before centrifugation (4 ºC, 1500 x *g*, 10 min). Supernatants were acidified with 1 M HCl and semi-purified by solid phase extraction (C18-E cartridges; 500 mg, 6 mL; Phenomenex). The eluate was dried under nitrogen, and the lipid residue was reconstituted in 100 µL ethanol for analysis. Eicosanoids were identified using multiple reaction monitoring (MRM) in the negative mode and quantified using commercially available standards (Cayman Chemical)

# Endocannabinoid analysis

Serum endocannabinoids and related N-acyl ethanolamines (NAEs) were quantified by UPLC/ESI-MS/MS, as previously described[2]. Briefly, serum samples were measured and diluted in 6 mL ice-cold 2:1 chloroform:methanol. Internal standards were added (20 ng AEA-*d*8 and 40 ng 2-AG-*d*8 (Cayman Chemical),) were added, and the samples incubated on ice for 30 min. Water (1 mL) was added to each sample, and samples were vortexed then centrifuged (4 ºC, 1500 x *g*, 10 min). The lower organic phase was removed and dried under nitrogen, and the lipid residue was reconstituted in 100 µL ethanol for analysis. Endocannabinoids and NAEs were identified by MRM in the positive mode and quantified using commercially available standards (Cayman Chemical).

# Ceramide analysis

Serum ceramides were analysed by UPLC/ESI-MS/MS. Extractions were performed as per endocannabinoid extractions above, except that the internal standard added was 50 pmol C25 Cer (Avanti Lipids, Alabaster, USA), and samples were reconstituted in 150 µL methanol with 0.1 % formic acid. Ceramides were identified by MRM in the positive mode and relative quantification performed against the internal standard as previously described[3].

# Protein preparation, FASP and phosphopeptide enrichment

Protein samples were lysed in 150mM NaCl, 1% Triton, 1mM PMSF, Complete Mini protease inhibitor cocktail, PhosStop, 20 mM Tris (pH7.5). For Luminex assays, the protein concentration was determined using a bradford assay, samples were then run on a SDS gel and stained with coomassie to check the concentration and determine the effect of haemolysis. Luminex assays were carried out according to the manufactures instructions (Bioplex 9plex MAPK kit). For FASP, protein samples were treated with 0.1%, 10mM DTT and then a further 2% SDS before heating at 95degrees for 5 min. FASP was carried out using a commercial kit (Protein digestion kit, Expedeon), 400ug of protein as used for FASP. Samples were acidified and dried down for proteome and phosphoproteome. Phosphopepetide enrichment was carried out using an automated protocol as described in[4], using MagRes Ti02/IMMAC beads (1:3 ratio) (ResynBiosciences). After enrichment, phosphopeptides were desalted using R3 media. Phosphoproteome analysis from LPS stimulated samples was carried out using the flow through lysate from the RNA preparation. The samples were lysed in BL buffer (Promega) and isopropanol.

Samples were then spun through the FASP column as described above. Peptides were quantified using a peptide assay (Thermo Scientific).

# Mass spectrometry

Digested samples were analysed by LC-MS/MS using an UltiMate^®^ 3000 Rapid Separation LC (RSLC, Dionex Corporation, Sunnyvale, CA) coupled to a Q Exactive HF (Thermo Fisher Scientific, Waltham, MA) mass spectrometer.

Peptide mixtures were separated using a multistep gradient from 95% A (0.1% FA in water) and 5% B (0.1% FA in acetonitrile) to 7% B at 1 min, 18% B at 58 min, 27% B in

72 min and 60% B at 74 min at 300 nL min^-1^, using a 75 mm x 250 μm i.d. 1.7 mM CSH M-Class C18, analytical column (Waters). The top 8 or 12 precursors were selected for fragmentation automatically by data dependant analysis during each cycle.

# Search settings

Raw MS files were processed with the MaxQuant software[5], using the integrated Andromeda search engine with FDR < 1% at peptide and protein level. Using a human protein database (UP000005640_9606). A reverse database for the decoy search was generated in MaxQuant. A minimum number of seven amino acids were required for peptide identification. For label-free protein quantification, the ‘Match Between Runs’ option was used with a window of 1 min. Variable and fixed modifications: (variable modification: acetylation (Protein-N terminus) and oxidation methionine (M), fixed modification: carbamidomethylation (C)). Phosphoproteome settings for variable and fixed modifications were variable modifications for

oxidation (methionine), acetylation (protein N-term), and pho (STY) and fixed modifications for carbamidomethyl (C). The MaxQuant LFQ algorithm was used for label free quantification. The Perseus framework was used to process the data. For both proteome and phosphoproteome, reverse and contaminant peptides were removed. A localisation probability of greater than 0.75 was used. Valid values were filtered on a per group basis. Missing values were imputed in Perseus[6].

# Additional statistical analysis

Proteome and phosphoproteome significance testing was carried out with the LIMMA package, using ~subject+time or using a T-test (with FDR) in the Perseus framework. Within subject effects were also tested using the duplicate correlation function. Heat maps were generated with Gplots, MD plots with LIMMA, all other plotting was carried out with ggplot2 (including Reactome pathway graphs). Luminex assays were analysed using a repeated measures ANVOA (TREATMENT*STATE*TIME+Error(SUBJECT). All other analysis was carried out in R. Phosphosites were retrieved Python, using Uniprot 1.3 and specific sequence information extracted using Pyfaidx (FASTA) Sequence logos were generated using the phoshosite website.

# Primers

cers1fwd gcc acc aca cac atc ttt cgg cers1 rev gga gca ggt aag cgc agt ag cers2fwd aga gtg ggc tct ctg gac g cers2rev cca ggg ttt atc cac agt gac

cers4fwd ctg tgg tac tgt tgt tgc atg ac cers4rev gcg cgt gta gaa gaa gac taa g cers5fwd tgg cca att atg cca gac gtg ag cers5rev ggt agg gcc caa taa tct ccc agc cers6fwd gca ttc aac gct ggt ttc gac cers6rev ttc aag aac cgg act ccg tag sptlc1fwd agg gtt cta tgg cac att tga tg sptlc1rev tgg ctt ctt cgg tct tca taa ac sptlc2fwd caa aga gct tcg gtg ctt cag sptlc2rev gaa tgt gtg cgc agg tag tct atc degs1fwd gaa tgg gtc tac acg gac cag degs1rev cga gaa gca tca tgg cta caa degs2fwd agc gac ttc gag tgg gtc ta degs2rev tcc ccg tac taa cca gca gg hprtfwd cac agg act aga aca cct gc hprtrev gct ggt gaa aag gac ctc t

# References

1. Pinto AM, Sanders TA, Kendall AC, Nicolaou A, Gray R, Al-Khatib H, et al. A comparison of heart rate variability, n-3 PUFA status and lipid mediator profile in age- and BMI-matched middle-aged vegans and omnivores. Br J Nutr. 2017 Mar; 117(5):669-685.
2. Felton SJ, Kendall AC, Almaedani AF, Urquhart P, Webb AR, Kift R, et al. Serum endocannabinoids and N-acyl ethanolamines and the influence of simulated solar UVR exposure in humans in vivo. Photochem Photobiol Sci. 2017 Apr; 16(4):564-574.
3. Kendall AC, Pilkington SM, Massey KA, Sassano G, Rhodes LE, Nicolaou A. Distribution of bioactive lipid mediators in human skin. J Invest Dermatol. 2015 Jun; 135(6):1510-1520.
4. Tape CJ, Worboys JD, Sinclair J, Gourlay R, Vogt J, McMahon KM, et al. Reproducible automated phosphopeptide enrichment using magnetic TiO2 and Ti- IMAC. Anal Chem. 2014 Oct; 86(20):10296-10302.
5. Tyanova S, Temu T, Cox J. The MaxQuant computational platform for mass spectrometry-based shotgun proteomics. Nat Protoc. 2016 Dec; 11(12):2301-2319.
6. Tyanova S, Cox J. Perseus: A Bioinformatics Platform for Integrative Analysis of Proteomics Data in Cancer Research. Methods Mol Biol. 2018; 1711:133-148.
