# Supplementary figures and images for: Rheumatoid arthritis reprograms circadian output pathways
Source: Arthritis Res Ther. 2019 Feb 6;21:47. doi: 10.1186/s13075-019-1825-y (PMC6366099; doi:10.1186/s13075-019-1825-y)

**Fig. S1**

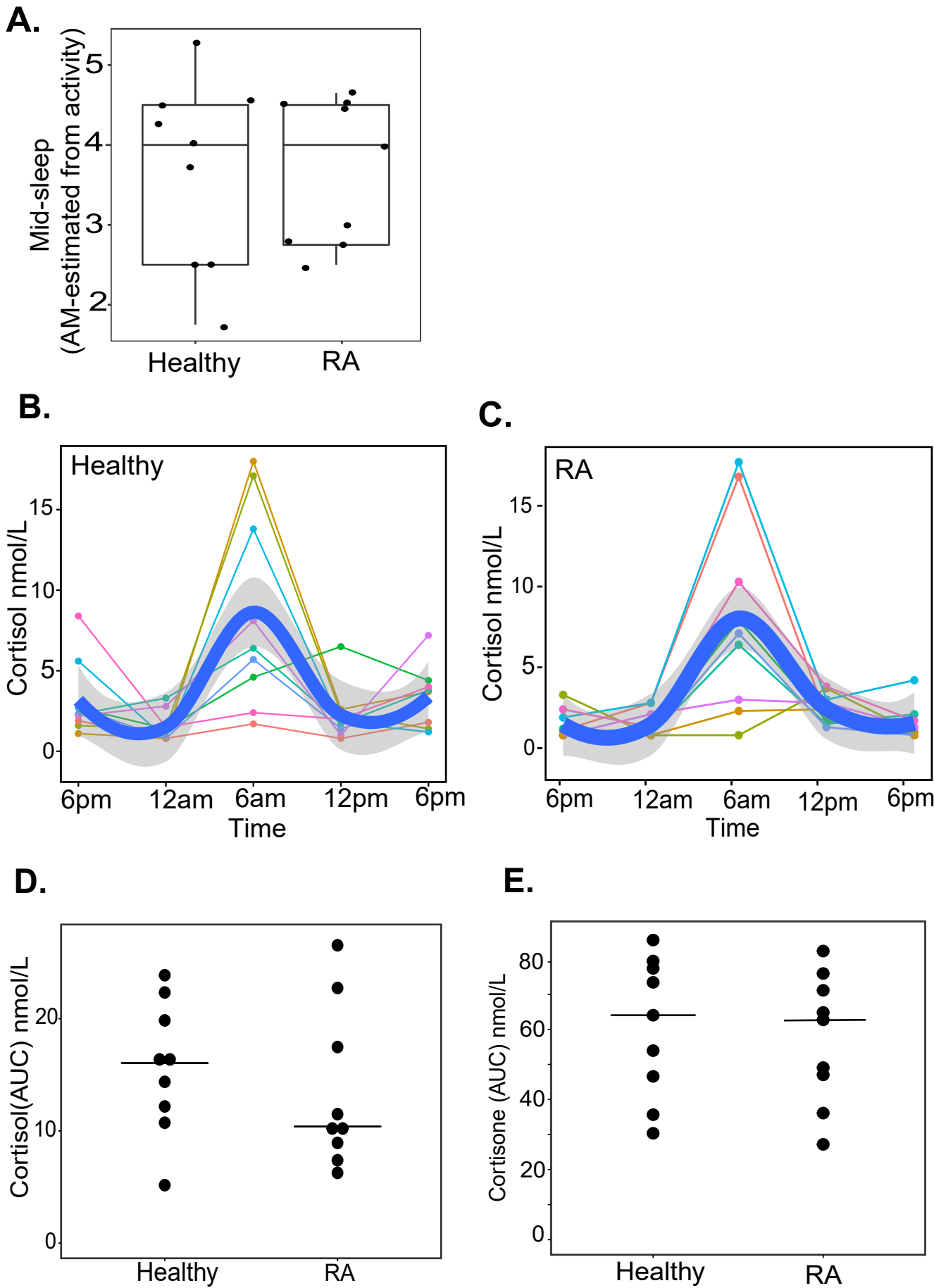

Supplement: Supplementary file 3 — Figure S1. Assessment of parameters of diurnal activity in RA patients. (A) Summary activity data for both healthy (n = 10) and RA patients (n = 10) was used to estimate the mid-rest phase which is plotted by clock time. (B) Salivary cortisol or cortisone levels were measured in healthy subjects (n = 9) and RA patients (n = 9) over 5 time points (from 6 pm at 6 hourly intervals). (D and E) Data expressed as area under the curve. (PDF 137 kb) [file 13075_2019_1825_MOESM3_ESM.pdf]

Fig.S'

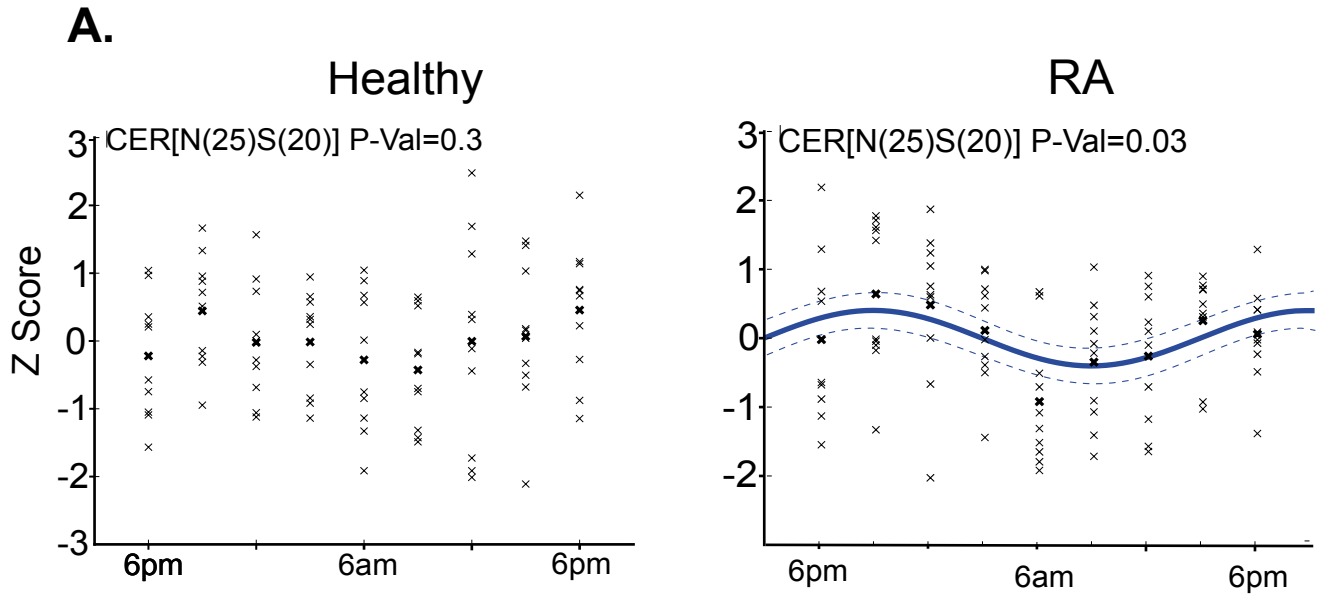

**B.**

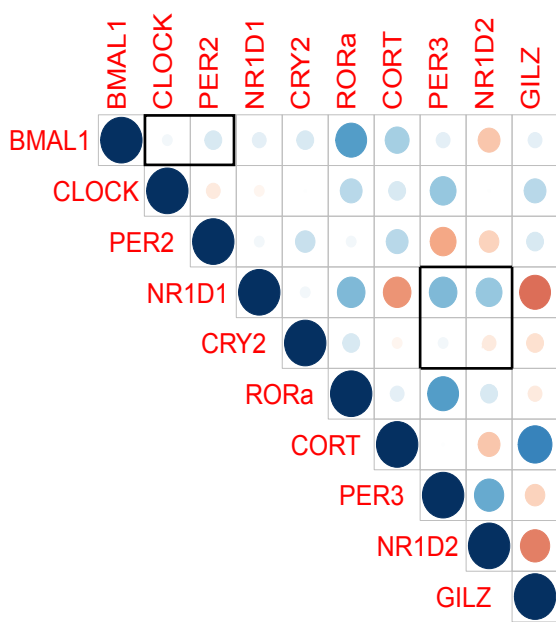

**C.**

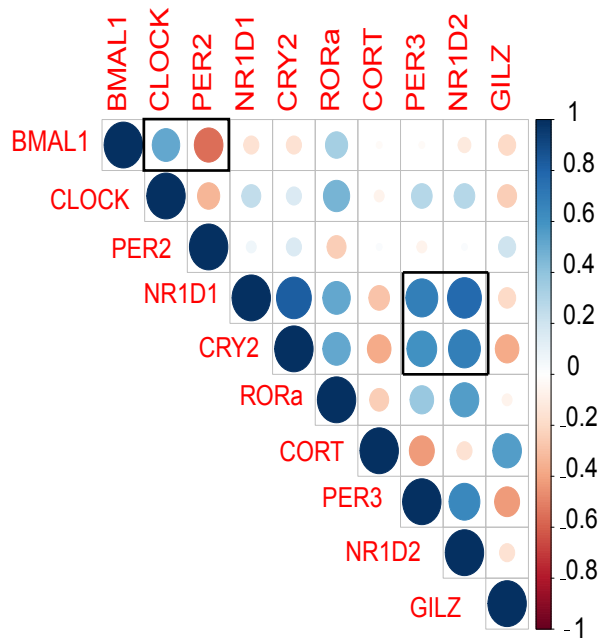

Supplement: Supplementary file 5 — Figure S5. (A) Healthy and (B) RA correlation analysis of circadian gene expression, and outputs of the circadian clock. The difference in gene expression (between 6 am and 6 pm) was calculated for each subject or patient, to estimate amplitude of oscillation. The difference between morning and evening salivary cortisone to determine circadian output pathway activity, and GILZ gene expression as a biomarker of cortisol action were also included in the correlation matrix. The circle diameter estimates strength of correlation, and the colour (red negative, and blue positive) the direction of correlation. (C) Circadian profile of CER[N(25)S(20)]in healthy and RA subjects (as identified in using a Gaussian process model). (PDF 290 kb) [file 13075_2019_1825_MOESM5_ESM.pdf]

Fig.S6

A.

Average arthritis score

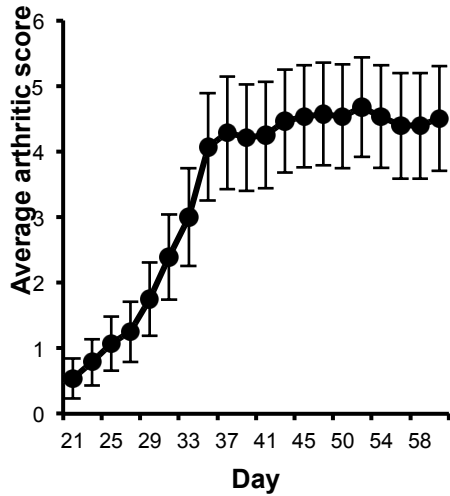

B.

Incidence

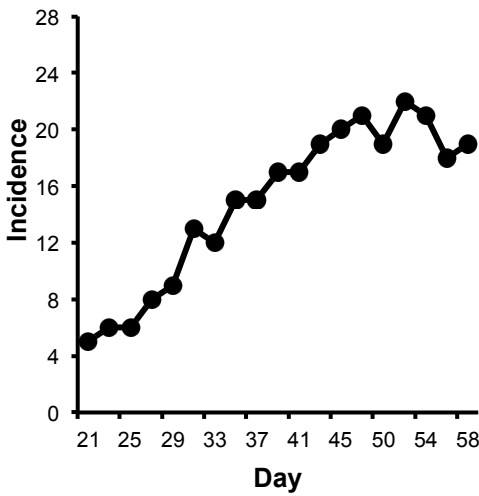

C.

Average hind paw thickness

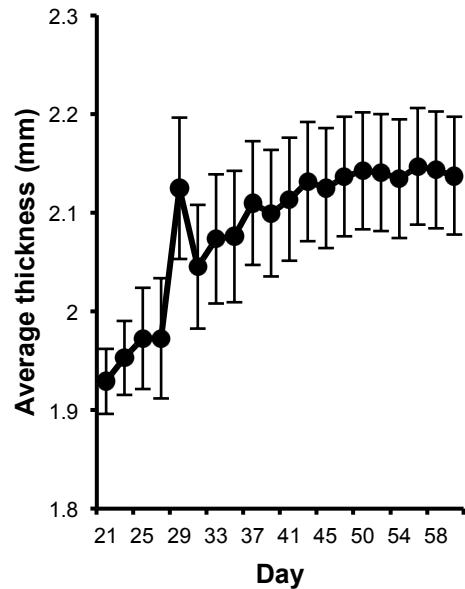

Supplement: Supplementary file 9 — Figure S6. Time course of arthritis induction in response to collagen immunisation in mice. The average arthritis score (A), incidence (B), and average hind paw thickness (C) were plotted against time following immunisation. Mean and standard error of the mean are shown. (PDF 183 kb) [file 13075_2019_1825_MOESM9_ESM.pdf]

Fig.S7

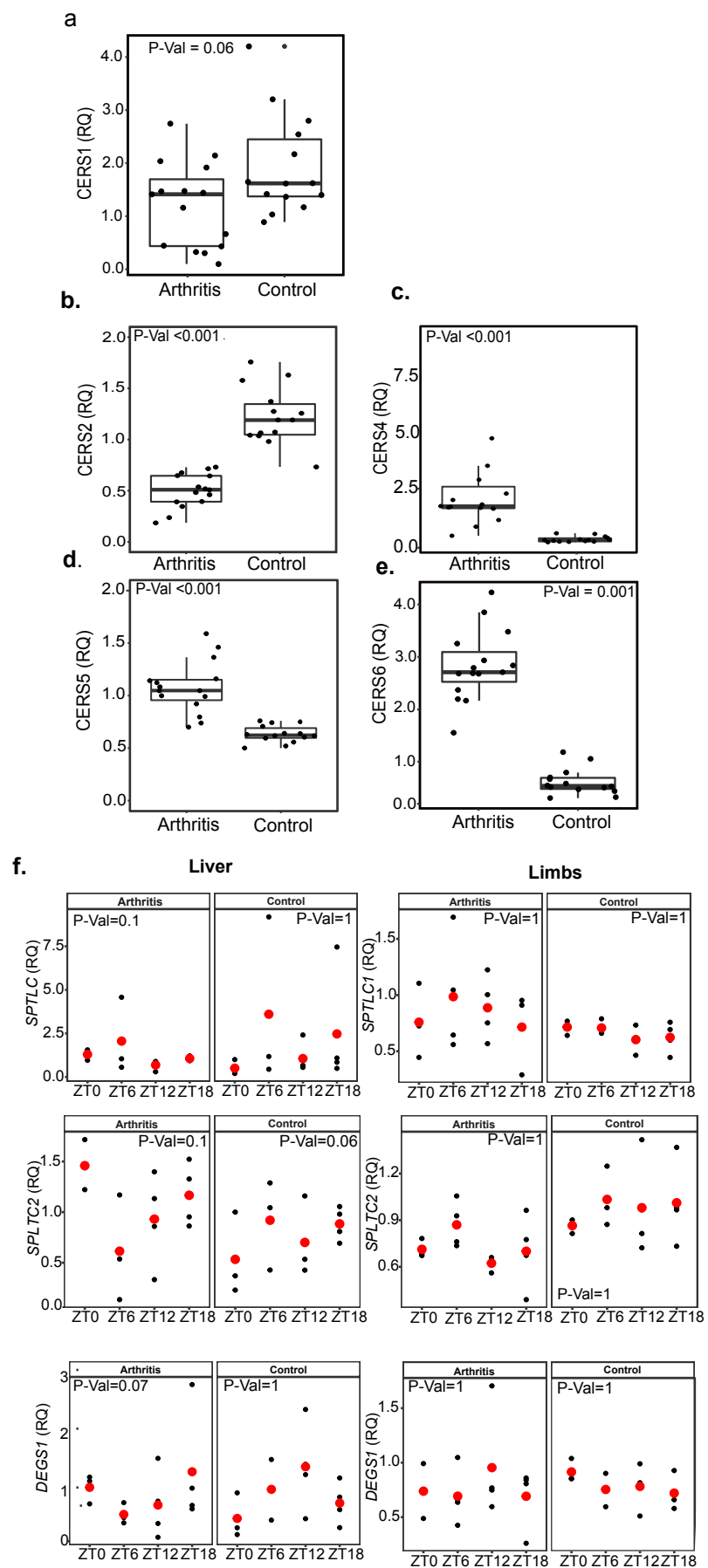

Supplement: Supplementary file 10 — Figure S7. Ceramide synthesis pathway gene expression analysis. (A-E) RNA was extracted from control and arthritic mouse limb tissue at six-hourly intervals (ZT0–18). Ceramide synthase gene expression was determined by qPCR. Mean gene expression data across time is shown as a boxplot. Changes in concentration over time were analysed by a Wilcoxon rank sum test (n = 14 control and 15 arthritis); exact P values are shown. (F) Ceramide synthetic pathway gene expression in liver and limb from CIA and control mice. Tissues were harvested at six hourly intervals as described. Gene expression for serine palmitoyltransferase long chain base subunit (SPLTC)1 and SPLTC2, dihydroceramide desaturase1 (DEGS1). JTK cycle was used to determine rhythmicity, adjusted P-values shown (control n = 13, arthritis n = 15). (PDF 796 kb) [file 13075_2019_1825_MOESM10_ESM.pdf]
