# Supplementary material for: Rheumatoid arthritis reprograms circadian output pathways
Source: Arthritis Res Ther. 2019 Feb 6;21:47. doi: 10.1186/s13075-019-1825-y (PMC6366099; doi:10.1186/s13075-019-1825-y)

Fig. S2

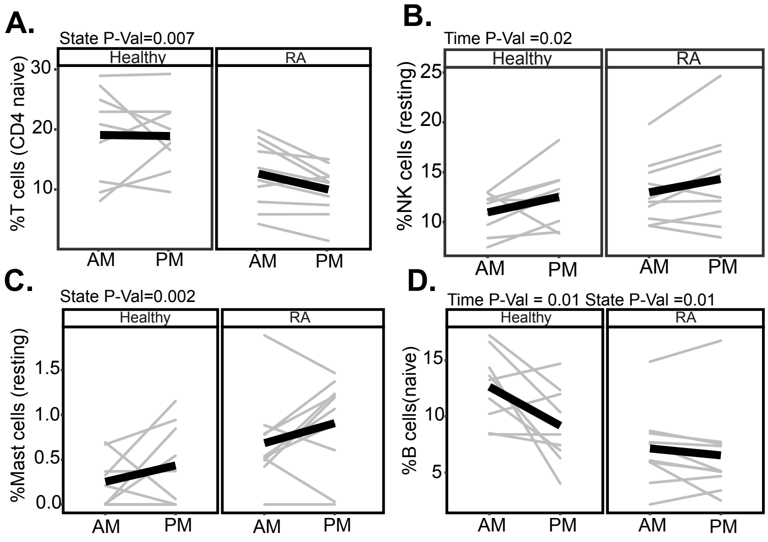

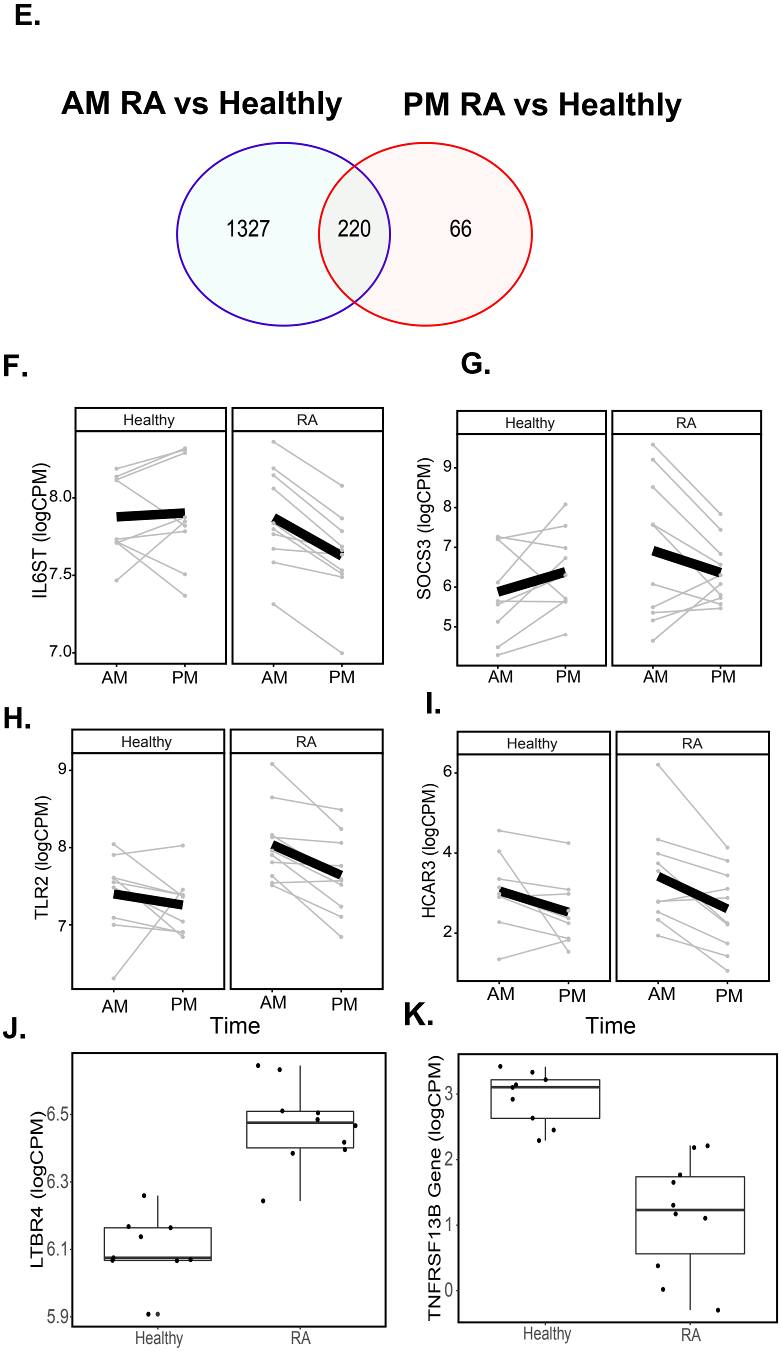

L. RA AM vs Healthy AM gene

Reactome pathway TLR4 cascade

SYMBOL	logFC	logCPM	LR	PValue	FDR
BIRC2	0.3258	6.4298	18.4610	0.0000	0.0023
IRAK4	0.2506	6.8487	13.7338	0.0002	0.0100
NOD2	0.7029	6.0349	13.6324	0.0002	0.0103
CD36	0.7708	8.5454	13.5198	0.0002	0.0105
PELI2	0.4303	5.3707	12.3410	0.0004	0.0145
ITGAM	0.5081	8.4576	11.6779	0.0006	0.0177
DUSP3	0.5481	5.9640	11.6088	0.0007	0.0180
S100A12	1.0028	6.2138	11.2498	0.0008	0.0201
IRAK3	0.6671	7.5830	10.8884	0.0010	0.0226
MAPK14	0.3921	7.0707	10.4565	0.0012	0.0256
LY96	0.7079	3.1553	9.2217	0.0024	0.0371
MAP3K7	0.1548	6.1184	8.8874	0.0029	0.0409
TLR2	0.6471	7.7056	8.7530	0.0031	0.0427
TBK1	0.3521	5.5614	8.7177	0.0032	0.0430
CHUK	0.2523	5.3874	8.7167	0.0032	0.0430

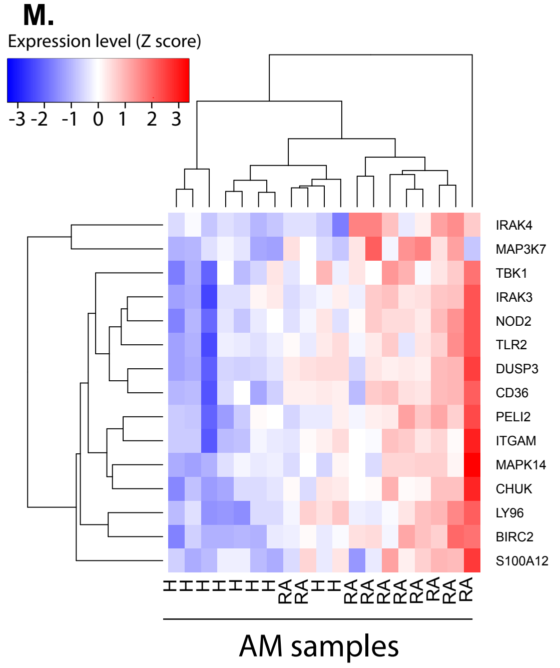

Supplement: Supplementary file 4 — Figure S2. Analysis of global gene expression in PBMLs from RA patients and healthy subjects. (A-D) Non-logged normalised CPM values were used for the analysis of PBMC cell populations by CIBERSORT. Estimated % of cell type for each subject (A) T lymphocytes, (B) NK cells, (C) Mast cells, and (D) B lymphocytes showed small differences between groups based on disease state (healthy or RA), or time of sampling (6 am or 6 pm). Significance was determined with a repeated measures ANOVA, significant p values for the overall effect of time (AM and PM), or state (Healthy or RA) regardless of time are shown (RA n = 10, healthy n = 9). (E) Venn diagrams were generated using all the significantly expressed genes in RA vs Healthy AM and RA vs Healthy PM. (F–K) Example genes from time-of-day RNA-SEQ analysis. Significant genes (AM RA vs Healthy) enriched in the Reactome pathway TL4 cascade are show as a table (with output from EdgeR) (L) and heatmap of expression level for each sample (M) (Expression level shown [Z score]. Samples were scaled by column; clustered by row and column.) (PDF 457 kb) [file 13075_2019_1825_MOESM4_ESM.pdf]
