# Supplementary material for: Rheumatoid arthritis reprograms circadian output pathways
Source: Arthritis Res Ther. 2019 Feb 6;21:47. doi: 10.1186/s13075-019-1825-y (PMC6366099; doi:10.1186/s13075-019-1825-y)

FigS3

A. RA AM LPS

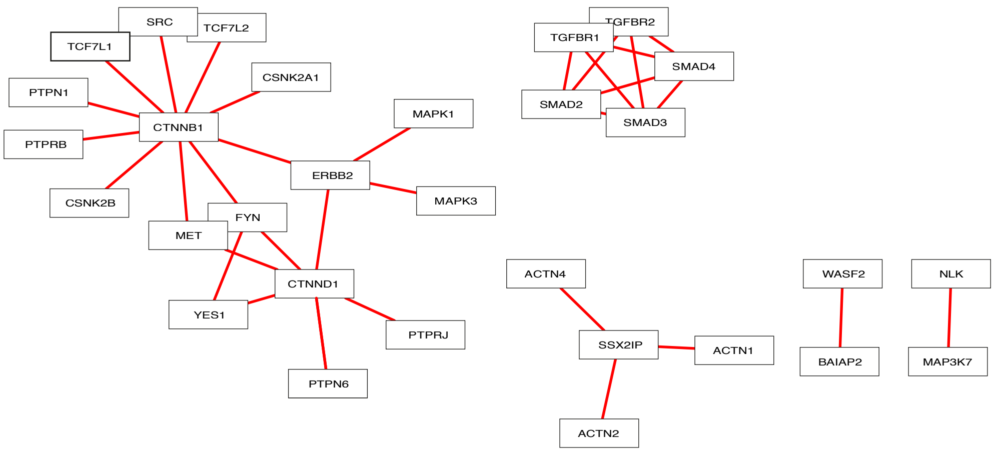

B. RA PM LPS

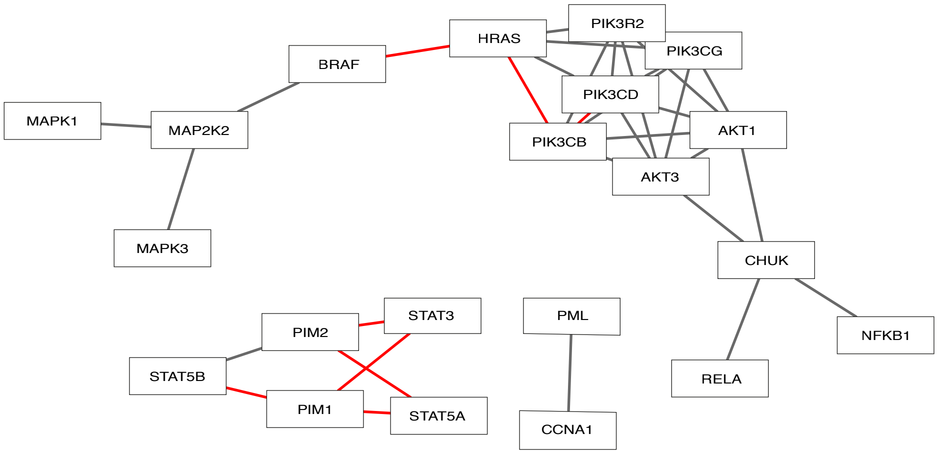

C. AM RA only LPS regulated gene network

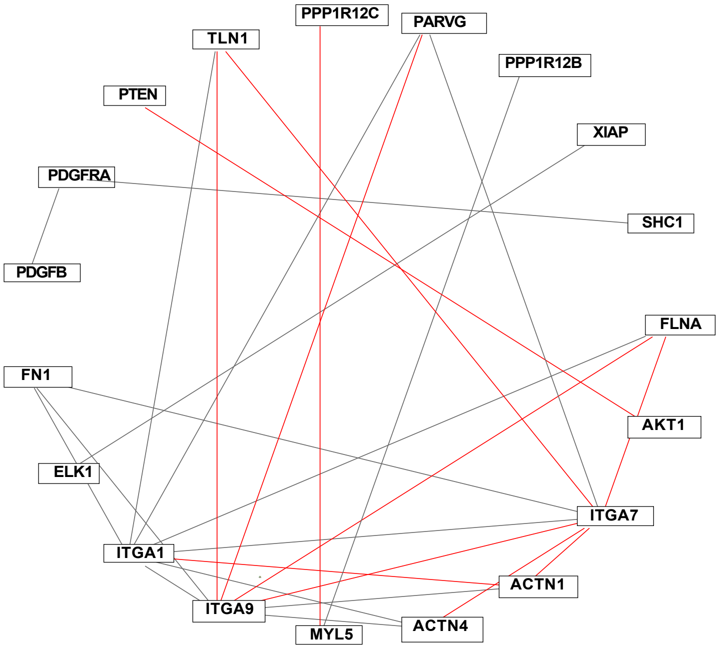

D. PM RA only LPS regulated gene network

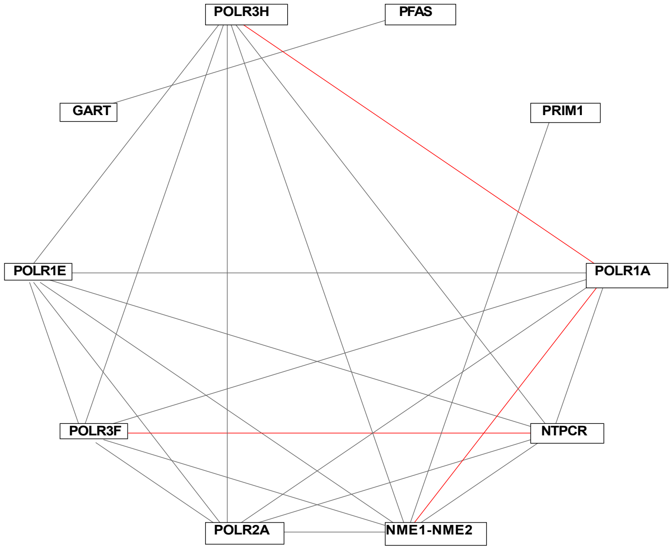

Supplement: Supplementary file 6 — Figure S3. Pathway analysis of AM and PM LPS regulated genes. Full networks for Fig. 4b. AM-RA LPS regulated genes were used to create network graphs using Edge set enrichment analysis (ESEA). (B) As (A) with genes regulated at 18:00. (C) Reactome pathways for 06:00 (LPS regulated Healthy vs RA) and (D) 18:00 (LPS regulated Healthy vs RA shown). (PDF 223 kb) [file 13075_2019_1825_MOESM6_ESM.pdf]
