# Supplementary material for: Rheumatoid arthritis reprograms circadian output pathways
Source: Arthritis Res Ther. 2019 Feb 6;21:47. doi: 10.1186/s13075-019-1825-y (PMC6366099; doi:10.1186/s13075-019-1825-y)

FigS4

A.

RA LPS AM DN-regulated

TF	NES	Targets	Motifs
RHOXF1	4.1	155	3
HMGB4	3.8	83	4
NFYB	3.8	39	1

RA LPS AM UP-regulated

TF	NES	Targets	Motifs
RXRA	4.6	44	2
SRF	4.2	121	7
DMRT2	3.8	68	3

B.

RA LPS PM DN-regulated

TF	NES	Targets	Motifs
YY1	4.1	267	3
GABPA	3.8	262	9
POLR3A	3.8	267	3

RA LPS PM UP-regulated

TF	NES	Targets	Motifs
CREB1	5.9	241	16
YY1	3.6	36	1
PPARG	3.6	115	4

C.

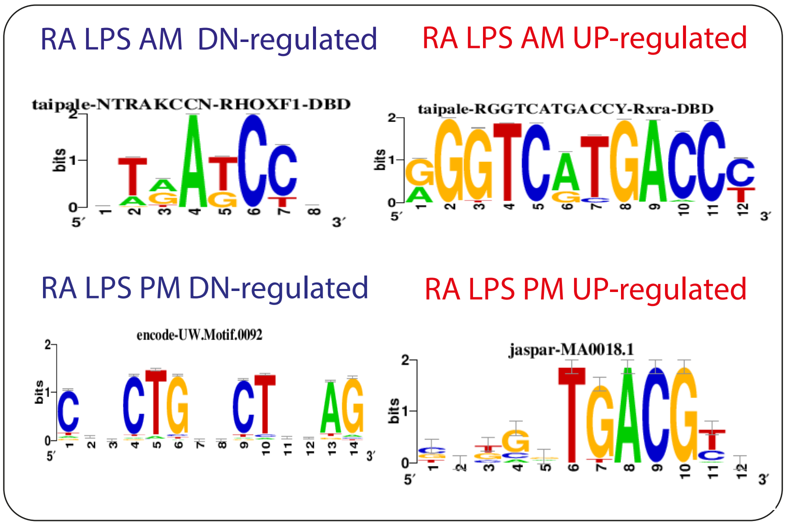

Supplement: Supplementary file 7 — Figure S4. Transcriptional regulators of AM and PM LPS regulated genes in RA. AM and PM LPS regulated genes were analysed using iRegulon (Cytoscape) A and B) Net enrichment scores were (NETs) were used to order the potential transcription factors. (C) Example motifs for the most significantly enriched targets identified in A and B. Normalised enrichment scores (NES) indicate a motif that covers a large proportion of the input genes (> 3, which corresponding to an FDR between 3 and 9%). (PDF 686 kb) [file 13075_2019_1825_MOESM7_ESM.pdf]
